# ANS: Aberrant Neurodevelopment of the Social Cognition Network in Adolescents with Autism Spectrum Disorders

**DOI:** 10.1371/journal.pone.0018905

**Published:** 2011-04-26

**Authors:** Yawei Cheng, Kun-Hsien Chou, Yang-Teng Fan, Ching-Po Lin

**Affiliations:** 1 Institute of Neuroscience, National Yang-Ming University, Taipei, Taiwan; 2 Department of Physical Medicine and Rehabilitation, National Yang-Ming University Hospital, Yilan, Taiwan; University of Queensland, Australia

## Abstract

**Background:**

Autism spectrum disorders (ASD) are characterized by aberrant neurodevelopment. Although the ASD brain undergoes precocious growth followed by decelerated maturation during early postnatal period of childhood, the neuroimaging approach has not been empirically applied to investigate how the ASD brain develops during adolescence.

**Methodology/Principal Findings:**

We enrolled 25 male adolescents with high functioning ASD and 25 typically developing controls for voxel-based morphometric analysis of structural magnetic resonance image. Results indicate that there is an imbalance of regional gray matter volumes and concentrations along with no global brain enlargement in adolescents with high functioning ASD relative to controls. Notably, the right inferior parietal lobule, a role in social cognition, have a significant interaction of age by groups as indicated by absence of an age-related gain of regional gray matter volume and concentration for neurodevelopmental maturation during adolescence.

**Conclusions/Significance:**

The findings indicate the neural correlates of social cognition exhibits aberrant neurodevelopment during adolescence in ASD, which may cast some light on the brain growth dysregulation hypothesis. The period of abnormal brain growth during adolescence may be characteristic of ASD. Age effects must be taken into account while measures of structural neuroimaging have been clinically put forward as potential phenotypes for ASD.

## Introduction

Autism spectrum disorders (ASD), a group of neurodevelopmental disorders, are characterized by impaired social reciprocity, communication difficulties, restricted interest and stereotyped behavior [1], [2]. Cascade failure of brain development could be the core deficit of ASD [3]. Based upon recent studies rethinking explanatory frameworks for developmental disorders, neuroimaging investigating brain development should help disentangle ASD pathogenesis [4], [5].

A number of studies reported that the ASD brain undergoes a period of precocious growth during early postnatal life, followed by a deceleration in age-related growth. The head circumference was found to be normal or below normal size at birth, followed by an increase in the rate of growth to the 84^th^ percentile by one year of age [6], [7], [8], [9], [10]. By 2 to 4 years of age, 90% of autistic toddlers have a 5% to 10% abnormal enlargement in the total brain volume. The 37% of these children have early developmental macrocephaly [6], [11], [12]. Thereafter, the brain shifts to abnormally slow growth. Infants with extreme brain growth fall into the severe end of the clinical spectrum with more extreme neuroanatomical abnormalities [7]. However, how this abnormal brain growth occurs during adolescence is not so clear [3], [6]. It is likely that adolescents within the mild end of clinical spectrum, such as, high functional autism and Asperger's syndrome, may not have dramatic brain growth during infancy.

Whether abnormal brain enlargement associated with ASD development results from disproportionately growth of the gray matter (GM) or white matter (WM) is controversial. The WM enlargement was reported to be larger than the GM from 1.5 to 4 years of age [6], [7], [10]. A 6% to 12% enlargement of GM collectively spans adolescence and adulthood [12], [13], [14]. It thus suggested that the GM enlargement might persist into adulthood in spite of the fact that the increase is disproportionately less than for WM early in life. However, the issue of the regional GM changes with age during adolescence in ASD needs more empirical researches.

Although previous structural magnetic resonance image (MRI) studies have reported cortical abnormalities in frontal, temporal, and parietal lobe, no clearly consistent pathology emerges for ASD. Children with autism had a significant reduction in total GM volume, particularly localized within fronto-striatal and parietal networks [15]. There is some evidence of greater GM volume in the dorsolateral prefrontal and medial frontal cortex [11], [16]. Findings are less consistent for the orbitofrontal cortex [17], [18]. Children with high-functional autism had smaller GM volume in predominantly fronto-pallidal regions, while children with Asperger's syndrome had less GM in mainly bilateral caudate and left thalamus [19]. Analysis of cortical shape identified that low-functional autism had prominent abnormal shapes in the pars opercularis whereas Asperger's syndrome had abnormalities in the intraparietal sulcus [20]. Cortical thickness showed an increase over the parietal and temporal cortex in children with ASD [18] whereas a decreased observed in the inferior frontal gyrus, inferior parietal lobule, and superior temporal sulcus in adults with ASD [21]. Particularly, the cortical thinning in the inferior frontal gyrus was correlated with their severities in social and communicational symptoms [21]. The cortical folding abnormalities in the intraparietal sulcus were correlated with age and social symptom severities in individuals with Asperger's syndrome [20]. Moreover, the autistic amygdala demonstrated an abnormal pattern of development with early enlargement in childhood followed by a reduction beginning from adolescence [22].

Here, to assess the convergent evidence on the developmental changes associated with neuroanatomical abnormalities, we performed a high-resolution anatomical MRI scanning with the analysis of whole-brain voxel-based morphometry (VBM) in adolescents with ASD and typically developing controls (TDC). VBM, a well-defined unbiased whole-brain technique on a voxel-by-voxel basis [23], has been crossly validated with region of interest (ROI) measurements, which enables to accurately identify subtle GM changes and to detect their associations with clinical symptoms [24], [25], [26]. For instance, the automated VBM technique was reported to detect a general trend of atrophy similar to the expertly labeled ROI measurements in Alzheimer's disease and semantic dementia. The estimated volume of temporal lobe from these two methods was highly correlated [26]. Here, this study applied VBM to probe GM volume and concentration changes specific to adolescents with ASD. Further, given that individuals with ASD were supposed to have brain growth dysmaturation during early childhood, we hypothesized that the abnormal neurodevelopmental maturation should extend to span adolescence. As compared with the controls, the global brain growth in adolescents with high functional autism and Asperger's syndrome may not be abnormal whereas their regional brain volumes and concentrations should have differential age-related changes.

## Materials and Methods

### Participants

Thirty adolescents with ASD and twenty-eight controls (TDC) participated in the study. Five individuals with ASD and three controls did not complete the MRI scan due to difficulties to tolerating the scanner environment and/or excessive head movements, the final sample consisted of twenty-five adolescents with ASD and twenty-five TDC. All participants were right-handed ethnic Chinese males with IQ>80 estimated by Wechsler Intelligence Scale for Children [27]. Adolescents with a co-morbid psychiatric or medical condition (e.g., epilepsy), history of head injury, or genetic disorders associated with autism (e.g., fragile X syndrome) were excluded. The ASD group had non-medicated adolescents with ASD aged 10–18 years, recruited from a community autism program. The TDC group had typically developing adolescents recruited from local schools and screened for major psychiatric illness using a structured parental interview. Every adolescent and his parents gave written informed consent for the study protocol approved by local Ethics Committee (National Yang-Ming University Hospital). This study was conducted in accordance with the Declaration of Helsinki.

### MRI acquisition

All subjects received scanning on the 1.5T MR scanner (Excite II; GE Medical Systems, Milwaukee, USA), equipped with an 8-channel head coil and with the whole brain T1 weighted 3D-FSPGR imaging protocol. The subject's head was aligned and immobilized with cushions inside the coil to avoid motion artifacts. If motion artifacts happened to the phase encoding direction of T1 images, we performed additional scans to obtain visibly acceptable images or withdrew the scans earlier. After scanning, we followed the semi-automatic approach [28] to evaluate the quality of T1 scans. Particularly for motion artifacts, individual tissue segment images were assigned to the rating of none, mild, moderate, or severe level. If any of the images were rated as the moderate or severe level, this individual's scanning would be excluded from VBM analysis.

We conducted a three-plane scan localizer to optimize the scanning slices parallel to the anterior–posterior commissure line. A three-dimensional fluid-attenuated inversion-recovery fast spoiled gradient recalled echo (FLAIR-FSPGR) was applied to obtain 124 contiguous T1-weighted (T1W) structural images: TR = 8.548 ms, TE = 1.836 ms, TI = 400 ms, flip angle = 15°, field of view (FOV)  = 26×26 cm, matrix size = 256×256, yielding the in-plane resolution of 1.02×1.02 mm, and the slice thickness = 1.5 mm.

### Data Processing for VBM

We conducted an optimized VBM protocol implemented within Matlab 6.5 (MathWorks, MA, USA) on statistical parametric mapping (SPM2; Wellcome Department of Imaging Neuroscience, London, UK) [23], [29], [30]. Preprocessing of the structural images followed the defined steps:

#### Manual preprocessing

The scans were manually reoriented with the inter-hemispheric gap parallel to the vertical axis of the view field and with the anterior-posterior commissure line parallel to the horizontal axis. The origin was manually set on the anterior commissure. The reorientation matrix using six parameters for rigid body transformation on the “Display” function was applied to original images.

#### Creation of group-specific templates and priors

To reduce any bias in template selection, a group template of images was established directly from the subject dataset. We generated the group-specific templates to reduce the error arising from registering individual images onto the template space. The group specific template was constructed with images of both groups (ASD vs. TDC) having exactly the sample size in order to reduce the risk of type II error. Considering brain differences between adults and adolescents, we used the pediatric T1W template of CCHMC2_boy (Cincinnati Children's Hospital Medical Center, Ohio, USA) associated with *a priori* probability maps. The T1W images were registered to the above template image by using 12 degrees of freedom affine transformation. All of the registered T1W images of the total fifty recruited subjects were averaged and smoothed with an 8-mm Gaussian kernel to generate the group template as the standard template for further analysis.

With the use of the CCHMC_boy priors, the normalized MRI images were segmented into cerebrospinal fluid (CSF), GM, and WM compartments. To correct for non-uniformity in image intensity, the SPM segmentation employs a mixture model cluster analysis to identify voxel intensities, which matches particular tissue types, in combination with *a priori* probabilistic knowledge of the spatial distribution of tissues derived from the GM, WM, and CSF of the prior probability images (i.e., the priors) [29], [30]. Next, the images were smoothed with an 8-mm kernel and averaged to obtain the group-specific CSF, GM, and WM priors for later segmentation of native scans. To improve segmentation quality, using Brain Extraction Tool (BET), available in FSL 4.0 (FMRIB Image Analysis Group, Oxford, UK), stripped the skull from the normalized scans with the fixed arguments (fractional intensity threshold = 0.4; vertical gradient = 0) [31].

#### Segmentation of the native scans and deviation from the optimized normalization parameters

Based on the group-specific CSF, GM, and WM priors, all of the original MRI native scans were segmented. This step involves an affine transformation of each scan to the group-specific T1 template with a subsequent back-projection into the native space, followed by an automated brain extraction procedure incorporating a segmentation step to remove non-brain tissue [26]. The extracted GM and WM images were then precisely normalized to the group-specific GM and WM template separately. The spatial normalization used the residual sum of squared differences as the matching criterion, and included affine transformations and linear combination of smooth basis functions modeling global non-linear shape differences [32].

#### Optimized normalization and segmentation

To reduce any contribution from non-brain voxels and to produce optimal spatial normalization of the GM and WM, the normalization parameters were applied to the original structural images in the native space. These normalized and skull-stripped structural images were then segmented into GM and WM partitions according to group-specific tissue priors.

#### Correction of volume change and final “clean” tissue modulated images

While introducing the Jacobian determinants derived from the spatial normalization, the non-linear spatial transformations modulate the regional differences of partitioned GM/WM from relative (concentration) into an absolute amount (volume) [23]. Using relative (concentration) tissue segment images, the binary GM mask of each participant was constructed to reduce any partial volume effect. Only the GM probability value larger than 0.3 (maximal value = 1) as well as greater than the corresponding WM and CSF probability value could pass the criteria of these individual masks. Modulated GM images were multiplied with the corresponding individual GM masks to obtain the final “clean” GM modulated images. All normalized, segmented, modulated, and masked images were smoothed with an 8 mm Gaussian Kernel before the voxel-wise group comparisons. In addition, to measure GM/WM concentration, the non-modulated GM/WM segments were smoothed with an 8 mm Gaussian kernel. GM/WM volume (modulated GM/WM segments) was acquired from unmodulated GM/WM segments after scaling amount of contraction during spatial normalization. The modulated tissue segments can preserve absolute tissue volume information. GM/WM concentration (unmodulated GM/WM segments) means the proportion of GM/WM segments to all tissue types within a voxel [33].

### Statistic Analysis

First, using all of the normalized, masked, modulated, and smoothed GM images, analysis of covariance (ANCOVA) in SPM2 was employed for the whole-brain voxel-wise comparison between groups (ASD vs. TDC) on GM volume. The individual tissue volumes were used as the dependent variable at each voxel in the standard space, co-varying for the total GM volume, the participant's age, and full-scale IQ. Using the smoothed non-modulated GM segments, ANCOVA for the whole-brain voxel-wise group comparison on GM concentration was performed while the global mean voxel value, the participant's age, and full-scale IQ being covariates. An uncorrected *P*-value<0.001 with a cluster size of more than 10 contiguous voxels was set to putatively detect significant differences between groups. Further, based on prior hypothesis, all of the significant clusters were required to perform Small Volume Correction (SVC), corresponding to anatomically defined regional masks in the Montreal Neurological Institute (MNI) template, to control multiple comparisons corrections. The corresponding masks were defined by the SPM extension– WFU-PickAtlas. Only the clusters passed through SVC-FWE (Family-Wise Error) *P*<0.05 were reported. Peak localization was determined from the MNI coordinates and identified by the Talairach and Tournoux atlas after a non-linear algorithm transformation (MRC Cognition and Brain Sciences Unit, Cambridge, UK).

Second, to elucidate any developmental change, we employed a whole-brain voxel-by-voxel analysis (SPM2) to test for the clusters showing the interaction of multiple linear regressions between group and age. The product of groups and age was included in the same model to predict the GM volume. Since our data was modulated, our model needs to include the global GM volume as a nuisance variable as well as the correction for IQ and global volumes [23], [26]. All of the significant clusters were required to pass through SVC-FWE *P*<0.05 for multiple comparisons corrections. In addition, to predict the GM concentration, the same statistical model for the interaction of age by groups was applied with the correction for IQ and the global mean voxel value. For the clusters showing a significant interaction, the “absolute GM content” (i.e., the sum of all voxel values of the cluster) and the “relative GM content” (i.e., the sum of all voxel values divided by the global GM) were further analyzed for the ASD and TDC using standard statistics software (SPSS, version 14.0.1)

## Results

### Demographics

The characteristics of the participants were listed in Table 1. These ASD individuals had the following diagnoses: 12 autism, 11 Asperger's syndrome, and 2 pervasive developmental disorders not otherwise specified, confirmed by experienced clinicians' evaluations using DSM-IV criteria as well as the Chinese version of Autism Diagnostic Interview-Revised (ADI-R) [34]. The subjects with ASD and the TDC were group-matched on age, full-scale IQ, and handedness.

**Table 1:** Participant Characteristics.

	ASD (n = 25)			TDC (n = 25)			*P*
	Mean	SD	range	Mean	SD	range	
**Age (years)**	13.7	2.5	10–18	13.5	2.1	11–18	0.726
**Full-scale IQ**	101.6	18.9	74–134	109.0	9.5	94–130	0.093
**Year of education**	7.0	2.3	4–11	6.5	1.2	5–9	0.878
**ADI-R**							
** Social interaction**	22.0	3.8	16–28	---	---	---	---
** Communication**	17.4	4.8	8–24	---	---	---	---
** Repetitive behavior**	5.2	1.3	1–7	---	---	---	---
** Total scores**	47.4	8.8	35–62	---	---	---	---

### Voxel-Based Morphometry

Neither the GM (ASD vs. TDC: 0.66±0.0 vs. 0.65±0.06 liters; *Z* = −0.18, *P* = 0.78) nor the WM (0.41±0.04 vs. 0.43±0.04 liters; *Z* = −0.18, *P* = 1.62) volume showed global differences between groups. Accordingly, the total intracranial volume of the ASD (1.07±0.07 liters) was similar to the TDC (1.08±0.09 liters) (*Z* = −0.67, *P* = 0.65). The gray-white absolute volume ratio was 1.60 for ASD and 1.53 for TDC, respectively.

**Figure 1:**
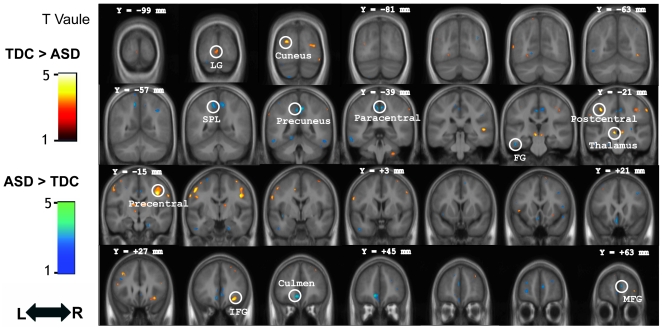
Direct comparison between groups controlling for age, IQ, and total gray matter volume. The gray matter regions showing significant group differences between individuals with ASD and TDC are rendered onto the averaged coronal images of the whole sample (N = 50) (Voxel threshold: uncorrected *P*<0.001). The y-coordinate for each coronal slice in the Montreal Neurological Institute space is given in millimeters. L: left, R: right. Abbreviations: LG, lingual gyrus; SPL, superior parietal lobule; FG, fusiform gyrus; IFG, inferior frontal gyrus; MFG, medial frontal gyrus.

Controlling for age, IQ, and total GM volume, the whole-brain voxel-wise group comparison between the ASD and the TDC (SVC-FWE *P*<0.05, *Z* score>3.15) on the GM volume revealed that the right inferior frontal gyrus, precentral gyrus, postcentral gyrus, cuneus, right superior temporal gyrus, and lingual gyrus had smaller GM volume in ASD. The regions that exhibited larger volumes in ASD were the cerebellum, paracental lobule, superior parietal lobule, medial frontal gyrus, fusiform gyrus, middle frontal gyrus, and subcallosal gyrus (Figure 1 and Table 2). The comparison between ASD subgroups (Autistic disorder vs. Asperger syndrome) showed that autistic disorder had less GM volume in the anterior cingulate, inusla, and superior temporal gyrus but Asperperger's syndrome had less in the medial frontal gyrus (Table S1, supplementary materials). On the GM concentration, the whole-brain voxel-wise group comparison between the ASD and the TDC (SVC-FWE *P*<0.05, *Z* score>3.15) showed that the left precentral gyrus was reduced in ASD. The regions with increased intensities in ASD were the anterior cingulate cortex, right paracentral lobule, right precuneus, right fusiform gyrus, right middle and inferior frontal gyrus (Table S2 and S3, supplementary materials). On the white matter volume, the left precentral gyrus was reduced but the right precuneurs and left aracentral lobule were increased in ASD (Table S4, supplementary materials).

**Table 2:** Group differences in regional gray matter volume.

	Peak coordinate			*Z*	Cluster size (mm^3^) (*P*<.001)
Anatomical location	X	y	z		
**TDC>ASD**					
Inferior frontal gyrus	32	30	−10	3.74	180
Precentral gyrus	44	−13	44	3.58	306
Postcentral gyrus	−39	−22	54	3.48	57
Cuneus	−26	−88	26	3.35	91
Thalamus	−8	−22	0	3.31	35
Lingual gyrus	−4	−96	−1	3.24	23
Superior temporal gyrus	52	−32	6	3.22	13
**ASD>TDC**					
Anterior cingulate	−8	42	−5	4.36	478
Paracentral lobule	3	−43	56	4.22	370
Superior parietal lobule	−13	−55	60	3.81	195
Precuneus	−4	−42	52	3.79	122
Medial frontal gyrus	7	61	15	3.72	59
Fusiform gyrus	−51	−25	−23	3.47	34
Middle frontal gyrus	42	19	45	3.28	17
Subcallosal gyrus	−5	18	−14	3.28	33

### Interaction of Age by Groups

The analytical results of the partial correlation with age within each group, respectively, were demonstrated on GM volume (Table 3). As for the regional GM volume growth with age during adolescence, the TDC had the posterior cingulate, right inferior parietal lobule, left fusiform gyrus and cuneus whereas the ASD had the right amygdala, right insula, right precentral gyrus, and bilateral superior temporal gyrus. Further, this pattern happened to the left superior, middle, and medial frontal gyrus in the TDC but to the bilateral inferior frontal gyrus in the ASD. As for the regional GM volume loss with age, the left middle occipital gyrus, bilateral inferior parietal lobule, bilateral inferior temporal gyrus, and left middle temporal gyrus were observed in the ASD, not in the TDC (Table S5, supplementary materials). Among the brain regions with significant correlation by age, the GM volume of right inferior parietal lobule (x 54, y −30, z 44) had a negative correlation with the social subscale of ADI-R (*r* = −47, *P* = 0.036). ASD adolescents with more severe social impairments were more likely to have reduced GM volume in the right inferior parietal lobule. Furthermore, the correlation analysis with age within each of ASD subgroups, separately, showed that the inferior parietal lobule with age-related GM volume reduction in autistic disorder were overlapped with that in Asperger's syndrome. Precuneus and superior parietal lobule were associated with age in autistic disorder whereas superior frontal gyrus and postcentral gyrus were in Asperger's syndrome (Table S6, supplementary materials).

**Table 3:** Regional gray matter volume positively correlated with age in each group.

	Peak coordinate			*Z*_≡_ score	Cluster size (mm^3^) (*P*<0.001)
Anatomical location	x	y	z		
**TDC**					
Posterior cingulate	22	−61	11	4.38	683
Superior frontal gyrus	−41	56	15	3.56	47
Middle frontal gyrus	−38	57	−7	4.19	110
Medial frontal gyrus	−3	42	24	3.34	28
Inferior parietal lobule	53	−30	44	3.67	34
Cuneus	−14	−73	11	3.57	233
Fusiform gyrus	−28	−45	−13	3.36	23
**ASD**					
Amygdala	18	−5	−11	3.59	1164
Insula	34	14	−5	3.33	246
Inferior frontal gyrus	46	24	4	4.77	375
	−54	19	22	3.49	119
Precental gyrus	49	9	8	3.85	403
Superior temporal gyrus	24	9	−33	3.72	218
	−25	10	−34	3.35	49

**Figure 2:**
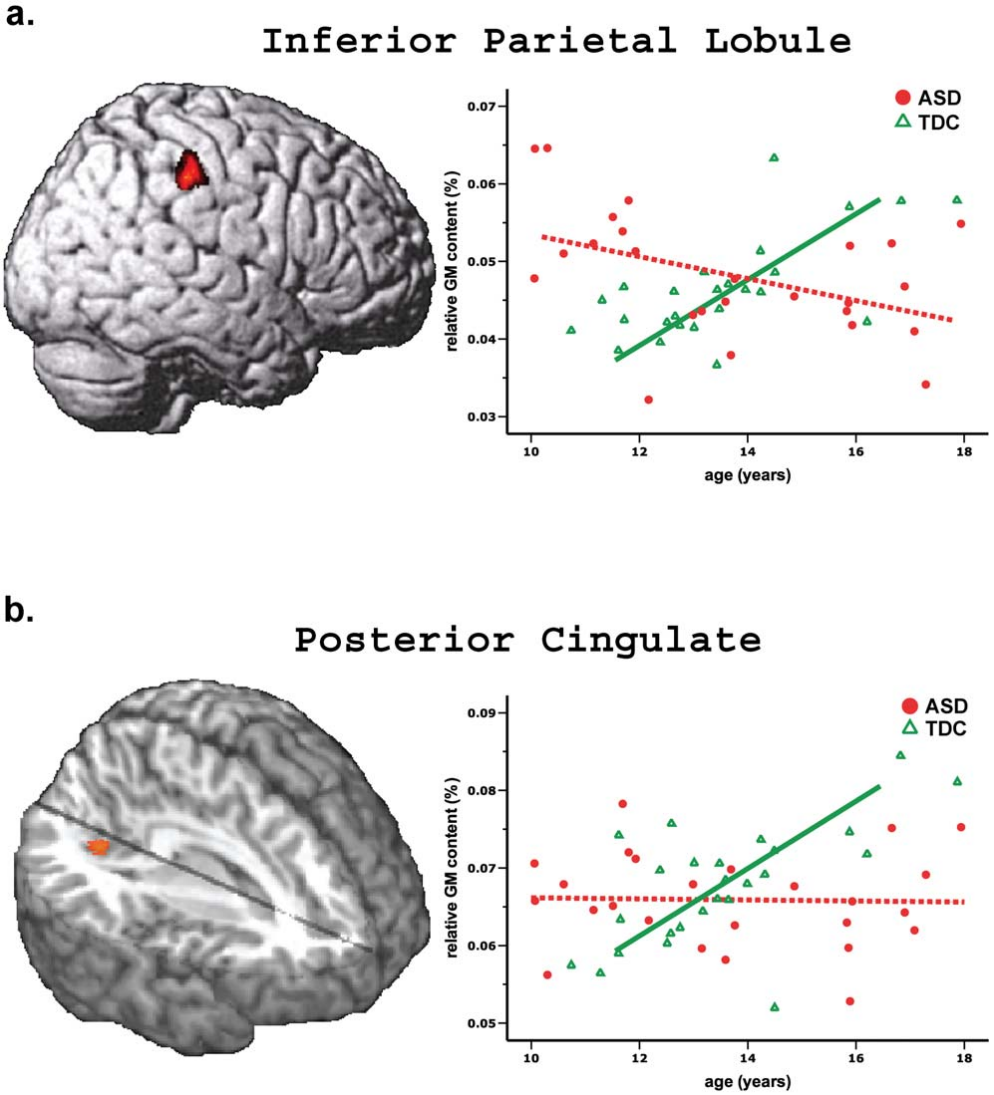
Significant interaction of age by group in the relative gray matter content. **a. Inferior parietal Lobule.** There is an age-related loss in the adolescents with ASD whilst an age-related gain in the TDC. **b. Posterior cingulate.** There is no age-related change in ASD but an age-related gain in the TDC.

Of note, the interaction of age by groups on GM volume reached significance in the right inferior parietal lobule (x 54, y −30, z 44) and posterior cingulate (x 20, y −63, z 12)(Table S7 and S8, supplementary materials). For the right inferior parietal lobule, the ASD showed an age-related loss of regional GM volume whilst the TDC showed an age-related gain. The relative GM content with age had a significantly positive correlation in the control (*β* = 0.652, *P* = 0.001) but a negative correlation in the ASD (*β* = −0.434, *P* = 0.034). Additionally, the posterior cingulate had a significant interaction (TDC: *β* = 0.672, *P* = 0.001; ASD: *β* = 0.021, *P* = 0.921) as indicated by the GM volume has a positive correlation with age in TDC instead of adolescents with ASD. Plotting the relative GM content of the right inferior parietal lobule and the posterior cingulate against age, respectively, was demonstrated in Figure 2. Additionally, the GM concentration of the left inferior parietal lobule (x −56, y −58, z 38) showed an age-related increase in the TDC group but an age-related decrease in the ASD group (Table S9, supplementary materials). No such interaction was found in the white matter volume (Table S10, supplementary materials).

## Discussion

The current findings indicate no significant difference in the global brain volume between the adolescents with ASD and typically developing controls. When age, IQ, and total GM volume are controlled, an imbalance of regional GM volumes and concentrations in participants with ASD appears compatible with previous reports [12], [13], [14], [15], [25], [35]. Notably, the regional GM volume of right inferior parietal lobule and posterior cingulate, a role in social cognition, in individuals with ASD failed to achieve an age-related gain for neurodevelopmental maturation during adolescence. The findings may shed some light on aberrant neurodevelopment extending from childhood into adolescence in ASD.

The imbalance of regional GM volumes and concentrations noted in the ASD adolescents could be compatible with previous studies [19], [35], [36], [37]. Volume enlargements in the medial prefrontal gyrus, cerebellum, and superior parietal lobule appear relatively consistently during ASD adolescence. Specifically, within the frontal lobe, the dorsolateral convexity shows significant overgrowth whereas the precental gyrus and orbitofrontal cortex are not really affected [16]. In addition, we found that ASD adolescents had a larger volume in the medial prefrontal cortex but a smaller volume in the lingual cortex. A handful of studies reported the frontal lobe having the greatest enlargements and the occipital lobes having the least [35], [36], [38]. This may suggest that the cortical areas most affected in ASD are phylogenetically and ontogenetically late-developing regions that are essential to complex human cognitive functions, such as executive function and social interaction.

To the best of our knowledge, the significant interaction of age by groups on regional GM volumes first demonstrates the neuodevelopmental change of social brain during adolescence of ASD. A large-scale longitudinal pediatric structural MRI study across childhood and adolescence reported nonlinear changes in the development of the cortical GM with involvement of a preadolescent increase followed by a post-adolescence decrease. The cortical GM volume of parietal lobe in cm^3^ peaked at age 12 in typically developing adolescents and then decline into early adulthood [39]. Here, with the use of VBM, the relative GM volume content (i.e., the sum of all voxel values divided by the global GM volume) and the regional GM concentration of the right inferior parietal lobule appeared an age-related gain at age 11 through 18 in TDC. Various methods for image analysis, including tissue segmentation (an artificial neural network vs. SPM) and brain parcellation strategy (region of interest vs. voxel-wise) may influence the findings of developmental curve on GM. Interestingly, such correlations with age changed in adolescents with ASD. That is, the right inferior parietal lobule in adolescents with ASD had rapid growth such that their GM volume and concentration reached adult size by age 14 whereas the TDC continued to show an increase. Similarly, the postmortem analysis of the amygdala in children with ASD had rapid growth to reach adult size by age 7 where as the controls continued to show an increase [22], [40]. The head circumference in children with ASD has been reported to be significantly smaller at birth and then increase disproportionately rapidly in the first year of life [7]. The integrity of white matter microstructure in paracentral lobule and superior frontal gyrus showed hyperplasia by age 13 and, thereafter, hypoplasia in adolescents with ASD [41]. The cortical folding abnormalities of intraparietal sulcus were associated with age through 7.5 to 18 years, particularly in children with Asperger's syndrome [20]. Here, the adolescents with autism had more age-related GM abnormalities in inferior parietal lobule whereas those with Asperger's syndrome had more in postcentral gyrus (Table S6, supplementary materials). Taken together, anomalous neurodevelopment in ASD subjects appears to extend from early postnatal period into adolescence. Abnormal regulation of brain growth in ASD may result in early overgrowth followed by abnormally slowed growth brain [42] during childhood and adolescence, a period when brain growth in normal subjects catches up. The period may herald a critical stage of development when individuals with ASD may fail to interact with the environment to guide selective synapse elimination over the normally wave of exuberant synaptogenesis.

It is important to note that the significant clusters with neurodevelopment abnormality among adolescents with ASD are located within the neural network involved in social cognition, including the right inferior parietal lobule and posterior cingulate. The right inferior parietal lobule is related to executive attention [43], sense of agency [44], and social perception [45]. The posterior cingulate is associated with emotional evaluation and perspective taking [46]. Further, the functional neuroimaging study reported that the subjects with ASD, reduced activation in these regions to integrate information concerning eye gaze, which, in turn, may cause their unable to understand other's mental status during social interaction [47]. Abnormalities in the anatomy and connectivity of the social brain systems may contribute to the behavioral phenotype in autism [15]. Here, the current findings showed autistic adolescents with more severe social impairments were more likely to show dysmaturation in right inferior parietal lobule. Adolescents with ASD have an aberrant development in the neural correlates responsible for social cognition.

When incorporated with sex differences in the regional GM volume of the inferior parietal lobule [48], [49], anomalous development of the right inferior parietal lobule during ASD adolescence noted here may reflect the extreme male brain theory of autism posited by Baron-Cohen [50]. The inferior parietal lobule, a neocortical region, is part of the heteromodal association cortex, which exhibits sexual dimorphism, particularly with regard to asymmetry. Male adults showed a leftward (left > right) asymmetry, with a less marked opposite asymmetry in females. Cortical thinning of the inferior parietal lobule of the ASD brain was correlated with their symptom severities [21]. Here we demonstrated that the male ASD adolescents shifted to an even more leftward asymmetry, as indicated by an age-related loss of regional GM volume in the right inferior parietal lobule.

Several limitations to this study need to be acknowledged. First, the spatial resolution of MRI scans may be limited by partial volume effects, which, in turn, may affect the GM and WM segmentation of the VBM procedures. Second, the range of IQ of individuals making up the groups was somewhat uneven (IQ: ASD 74 to 134, control 94 to 130) in spite of no significant differences between groups (*P* = 0.093). IQ can be a potential variable to influence GM development [51], which could have introduced some variability into the size of some of the structures that we hypothesized as differing change with age between groups. Thus, we statistically treated the IQ effects as a covariate in the main analysis. Finally, the lack of inclusion of female participants, in spite of advantages in terms of sample homogeneity, may limit the generalization of our results to the entire population with ASD. This may not be the optimal design, however, and further studies that recruitment of females, larger sample size, and longitudinal design are warranted.

The current MRI study with the use of VBM points out the presence of aberrant development in the social cognition network during adolescence in ASD. The findings can cast some light on the brain growth dysregulation hypothesis and the social brain hypothesis of autism, and may help partially explain why previous reports for ASD neuroanatomy are usually inconsistent. Even with good age matching, like our study, the age could impact upon the results driven by group comparison designs. Moreover, we empirically applied diffusion tensor imaging to demonstrate age-related disconnectivity on the integrity of white matter microstructure in adolescents with ASD [40]. One recent study reported that adolescents with ASD show accelerated age-related temporal and parietal cortical thinning [52]. Taken together, the period of abnormal brain growth during adolescence may be characteristic of ASD. Age effects must be taken into account while measures of structural neuroimaging have been clinically put forward as potential phenotypes for ASD.

## Supporting Information

Table S1 **Subgroup differences in gray matter volume (control vs. Autism vs. Asperger's syndrome).**
(DOC)

Table S2 **Group differences in regional gray matter concentration.**
(DOCX)

Table S3 **Subgroup differences in gray matter concentration (control vs. Autism vs. Asperger's syndrome).**
(DOCX)

Table S4 **Group differences in white matter volume.**
(DOCX)

Table S5 **Regional gray matter volume negatively correlated with age in each group.**
(DOCX)

Table S6 **Regional gray matter volume negative correlated with age in each ASD subgroup.**
(DOCX)

Table S7 **Interaction effects of age by group in regional gray matter volume.**
(DOCX)

Table S8 **Interaction effects of age by subgroup (Autism vs. Asperger's syndrome) in regional gray matter volume.**
(DOCX)

Table S9 **Interaction effects of age by group in regional gray matter concentration.**
(DOCX)

Table S10 **Interaction effects of age by group in regional white matter volume.**
(DOCX)
