# Supplementary material for: ANS: Aberrant Neurodevelopment of the Social Cognition Network in Adolescents with Autism Spectrum Disorders
Source: PLoS One. 2011 Apr 26;6(4):e18905. doi: 10.1371/journal.pone.0018905 (PMC3082537; doi:10.1371/journal.pone.0018905)
Supplement: Table S1 — Subgroup differences in gray matter volume (control vs. Autism vs. Asperger's syndrome). (DOC) [file pone.0018905.s001.doc]

**Table S1**: Subgroup differences in gray matter volume (control vs. Autism vs. Asperger’s syndrome)

|  | **Peak coordinate** | | | ***Z*** | **Cluster size (mm^3^) (*P* < 0.001)** |
| --- | --- | --- | --- | --- | --- |
| **Anatomical location** | **x** | **y** | **z** |  |  |
| **TDC > Asperger** |  |  |  |  |  |
| **Cuneus** | -26 | -89 | 26 | 4.1 | 228 |
| **Middle occipital gyrus** | 32 | -89 | 18 | 3.96 | 143 |
| **Middle frontal gyrus** | 36 | 7 | 56 | 3.71 | 96 |
| **Precentral gyrus** | -39 | -20 | 53 | 3.69 | 152 |
| **Thalamus** | -7 | -18 | 3 | 3.5 | 97 |
| **Precentral gyrus** | 41 | -14 | 57 | 3.43 | 78 |
| **Precentral gyrus** | -62 | 4 | 35 | 3.37 | 42 |
| **Superior temporal gyrus** | 51 | -33 | 8 | 3.36 | 37 |
| **Inferior frontal gyrus** | 34 | 30 | -10 | 3.22 | 30 |
| **Thalamus** | 6 | -19 | 2 | 3.19 | 15 |
| **TDC > Autism** |  |  |  |  |  |
| **Lingual gyrus** | -27 | -80 | -6 | 3.48 | 102 |
| **Inferior parietal lobule** | -51 | -39 | 24 | 3.19 | 10 |
| **Asperger > TDC** |  |  |  |  |  |
| **Anterior cingulate** | -10 | 42 | -6 | 5 | 862 |
| **Anterior cingulate** | 3 | 36 | -3 | 3.9 | 330 |
| **Inferior temporal gyrus** | -52 | -23 | -22 | 3.77 | 24 |
| **Precuneus** | 8 | -46 | 57 | 3.6 | 433 |
| **Insula** | 42 | -15 | -2 | 3.59 | 43 |
| **Middle frontal gyrus** | 38 | 39 | 11 | 3.47 | 71 |
| **Precuneus** | -4 | -42 | 50 | 3.27 | 15 |
| **Autism > TDC** | | | | | |
| **Medial frontal gyrus** | -5 | 49 | 24 | 3.6 | 46 |
| **Paracentral lobule** | 3 | -44 | 56 | 3.55 | 38 |
| **Asperger syndrome > Autism** | | | | | |
| **Insula** | 42 | -16 | -1 | 3.95 | 190 |
| **Anterior cingulate** | -13 | 42 | -5 | 3.69 | 106 |
| **Lingual gyrus** | 18 | -67 | 3 | 3.62 | 58 |
| **Inferior parietal lobule** | -45 | -27 | 27 | 3.39 | 36 |
| **Lingual gyrus** | -22 | -84 | -10 | 3.38 | 43 |
| **Anterior cingulate** | 6 | 34 | -8 | 3.36 | 144 |
| **Superior temporal gyrus** | -50 | -32 | 10 | 3.27 | 43 |
| **Postcentral gyrus** | -51 | -21 | 30 | 3.25 | 10 |
| **Parahippocampal gyrus** | 24 | -26 | -7 | 3.24 | 18 |
| **Autism > Asperger syndrome** | | | | | |
| **Middle frontal gyrus** | 37 | 7 | 54 | 3.67 | 168 |
| **Superior occipital gyrus** | -29 | -91 | 26 | 3.32 | 31 |
| **Medial frontal gyrus** | 6 | 38 | 32 | 3.28 | 15 |
