# Supplementary material for: ANS: Aberrant Neurodevelopment of the Social Cognition Network in Adolescents with Autism Spectrum Disorders
Source: PLoS One. 2011 Apr 26;6(4):e18905. doi: 10.1371/journal.pone.0018905 (PMC3082537; doi:10.1371/journal.pone.0018905)
Supplement: Table S2 — Group differences in regional gray matter concentration. (DOCX) [file pone.0018905.s002.docx]

**Table S2: Group differences in regional gray matter concentration**

|  | **Peak coordinate** | | | ***Z*** | **Cluster size (mm^3^) (*P* < 0.001)** |
| --- | --- | --- | --- | --- | --- |
| **Anatomical location** | **x** | **y** | **z** |  |  |
| **TDC > ASD** |  |  |  |  |  |
| **Left precentral gyrus** | **-29** | **-16** | **53** | **3.41** | **48** |
| **ASD > TDC** |  |  |  |  |  |
| **Anterior cingulate** | **-9** | **42** | **-6** | **5.05** | **560** |
| **Paracentral lobule** | **4** | **-14** | **47** | **4.38** | **359** |
| **Precuneus** | **7** | **-45** | **55** | **4.24** | **291** |
| **Medial frontal gyrus** | **-10** | **13** | **44** | **4.17** | **107** |
| **Middle frontal gyrus** | **44** | **18** | **45** | **3.79** | **49** |
| **Inferior frontal gyrus** | **52** | **16** | **14** | **3.74** | **53** |
| **Inferior temporal gyrus** | **56** | **-1** | **-31** | **3.67** | **109** |
| **Fusiform gyrus** | **30** | **-38** | **-18** | **3.45** | **37** |
