# Supplementary material for: ANS: Aberrant Neurodevelopment of the Social Cognition Network in Adolescents with Autism Spectrum Disorders
Source: PLoS One. 2011 Apr 26;6(4):e18905. doi: 10.1371/journal.pone.0018905 (PMC3082537; doi:10.1371/journal.pone.0018905)
Supplement: Table S3 — Subgroup differences in gray matter concentration (control vs. Autism vs. Asperger's syndrome). (DOCX) [file pone.0018905.s003.docx]

**Table S3: Subgroup differences in gray matter concentration (control vs. Autism vs. Asperger’s syndrome)**

|  | **Peak coordinate** | | | ***Z*** | **Cluster size (mm^3^) (*P* < 0.001)** |
| --- | --- | --- | --- | --- | --- |
| **Anatomical location** | **x** | **y** | **z** |  |  |
| **TDC > Asperger** |  |  |  |  |  |
| **Inferior parietal lobule** | **64** | **-41** | **51** | **3.91** | **26** |
| **Middle temporal gyrus** | **53** | **-32** | **7** | **3.44** | **29** |
| **Cerebellum, posterior lobe** | **-27** | **-79** | **-42** | **3.25** | **36** |
| **TDC > Autism** |  |  |  |  |  |
| **Lentiform nucleus** | **-19** | **-16** | **-7** | **2.89** | **23** |
| **Asperger > TDC** |  |  |  |  |  |
| **Anterior cingulate** | **-11** | **43** | **-5** | **5.45** | **566** |
| **Middle frontal gyrus** | **42** | **41** | **15** | **4.14** | **108** |
| **Paracentral lobule** | **5** | **-12** | **46** | **4.06** | **151** |
| **Insula** | **-38** | **8** | **17** | **3.78** | **53** |
| **Medial frontal gyrus** | **-9** | **33** | **36** | **3.74** | **153** |
| **Anterior cingulate** | **6** | **35** | **-4** | **3.70** | **119** |
| **Inferior temporal gyrus** | **53** | **-9** | **-34** | **3.66** | **233** |
| **Precuneus** | **11** | **-52** | **58** | **3.57** | **64** |
| **Postcentral gyrus** | **-47** | **-24** | **27** | **3.44** | **29** |
| **Lingual gyrus** | **-20** | **-67** | **0** | **3.25** | **20** |
| **Parahippocampus** | **-27** | **-19** | **-24** | **3.22** | **30** |
| **Autism > TDC** |  |  |  |  |  |
| **Precuneus** | **-25** | **-80** | **52** | **4.83** | **744** |
| **Medial frontal gyrus** | **-10** | **14** | **44** | **4.26** | **101** |
| **Paracentral lobule** | **5** | **-44** | **55** | **3.83** | **69** |
| **Inferior frontal gyrus** | **51** | **15** | **14** | **3.80** | **44** |
| **Caudate nucleus** | **-12** | **6** | **14** | **3.52** | **64** |
| **Cerebellum, anterior lobe** | **4** | **-52** | **-18** | **3.35** | **142** |
| **Asperger > Autism** |  |  |  |  |  |
| **Middle frontal gyrus** | **44** | **41** | **16** | **3.94** | **91** |
| **Amygdala** | **-21** | **-8** | **-8** | **3.66** | **363** |
| **Postcentral gyrus** | **-45** | **-27** | **28** | **3.64** | **35** |
| **Insula** | **-37** | **7** | **16** | **3.56** | **23** |
| **Medial frontal gyrus** | **-10** | **42** | **27** | **3.47** | **18** |
| **Lingual gyrus** | **17** | **-68** | **4** | **3.32** | **17** |
| **Caudate nucleus** | **7** | **2** | **0** | **3.28** | **41** |
| **Autism > Asperger** |  |  |  |  |  |
| **Cerebellum, posterior lobe** | **-27** | **-79** | **-54** | **3.77** | **181** |
| **Inferior parietal lobule** | **64** | **-40** | **51** | **3.63** | **18** |
