# Supplementary material for: ANS: Aberrant Neurodevelopment of the Social Cognition Network in Adolescents with Autism Spectrum Disorders
Source: PLoS One. 2011 Apr 26;6(4):e18905. doi: 10.1371/journal.pone.0018905 (PMC3082537; doi:10.1371/journal.pone.0018905)
Supplement: Table S4 — Group differences in white matter volume. (DOCX) [file pone.0018905.s004.docx]

**Table S4: Group differences in white matter volume**

|  | **Peak coordinate** | | | ***Z*** | **Cluster size (mm^3^) (*P* < 0.001)** |
| --- | --- | --- | --- | --- | --- |
| **Anatomical location** | **x** | **y** | **z** |  |  |
| **TDC > ASD** |  |  |  |  |  |
| **Left precentral gyrus** | -29 | -16 | 53 | 3.41 | 48 |
| **ASD > TDC** |  |  |  |  |  |
| **Midbrain** | -8 | -27 | -3 | 5.12 | 1426 |
| **Paracentral lobule** | -19 | -43 | 57 | 3.72 | 276 |
| **Precuneus** | 19 | -54 | 45 | 3.18 | 18 |
