# Supplementary material for: ANS: Aberrant Neurodevelopment of the Social Cognition Network in Adolescents with Autism Spectrum Disorders
Source: PLoS One. 2011 Apr 26;6(4):e18905. doi: 10.1371/journal.pone.0018905 (PMC3082537; doi:10.1371/journal.pone.0018905)
Supplement: Table S5 — Regional gray matter volume negatively correlated with age in each group. (DOCX) [file pone.0018905.s005.docx]

**Table S5: Regional gray matter volume negatively correlated with age in each group**

|  | **Peak coordinate** | | | ***Z*_≡_ score** | **Cluster size (mm^3^) (*P* < 0.001)** |
| --- | --- | --- | --- | --- | --- |
| **Anatomical location** | **x** | **y** | **Z** |  |  |
| **TDC** |  |  |  |  |  |
| **Supramarginal gyrus** | **63** | **-57** | **40** | **3.45** | **52** |
| **Angular gyrus** | **-47** | **-76** | **31** | **3.37** | **71** |
| **ASD** |  |  |  |  |  |
| **Middle occipital gyrus** | **-57** | **-69** | **8** | **3.57** | **613** |
| **Inferior parietal lobule** | **48** | **-35** | **50** | **3.84** | **189** |
|  | **-63** | **-30** | **23** | **3.43** | **186** |
| **Inferior temporal gyrus** | **56** | **-62** | **-5** | **3.46** | **54** |
|  | **-56** | **-65** | **-1** | **3.40** | **613** |
| **Middle temporal gyrus** | **-49** | **-74** | **18** | **3.29** | **114** |
