# Supplementary material for: ANS: Aberrant Neurodevelopment of the Social Cognition Network in Adolescents with Autism Spectrum Disorders
Source: PLoS One. 2011 Apr 26;6(4):e18905. doi: 10.1371/journal.pone.0018905 (PMC3082537; doi:10.1371/journal.pone.0018905)
Supplement: Table S6 — Regional gray matter volume negative correlated with age in each ASD subgroup. (DOCX) [file pone.0018905.s006.docx]

**Table S6: Regional gray matter volume negative correlated with age in each ASD subgroup**

|  | **Peak coordinate** | | | ***Z*_≡_ score** | **Cluster size (mm^3^) (*P* < 0.001)** |
| --- | --- | --- | --- | --- | --- |
| **Anatomical location** | **X** | **y** | **z** |  |  |
| **Autistic disorder** |  |  |  |  |  |
| Inferior parietal lobule | -53 | -60 | 54 | 4.98 | 342 |
|  | 59 | -54 | 49 | 3.39 | 11 |
| Superior parietal lobule | 45 | -59 | 62 | 3.490 | 189 |
|  | -31 | -52 | 54 | 3.54 | 29 |
| Lingual gyrus | 21 | -72 | -4 | 3.57 | 97 |
| Precuneus | 35 | -85 | 46 | 3.51 | 41 |
|  | -25 | -73 | 40 | 3.42 | 26 |
| **Asperger syndrome** |  |  |  |  |  |
| Postcentral gyrus | 59 | -23 | 50 | 4.55 | 466 |
|  | -56 | -35 | 50 | 3.96 | 197 |
| Inferior parietal lobule | -67 | -28 | 26 | 4.29 | 109 |
|  | -63 | -54 | 47 | 3.84 | 65 |
| Superior temporal gyrus | 37 | -54 | 13 | 4.06 | 81 |
| Angular gyrus | 47 | -75 | 31 | 3.90 | 354 |
| Precentral gyrus | 35 | -27 | 70 | 3.89 | 61 |
| Middle frontal gyrus | 35 | 7 | 46 | 3.79 | 30 |
|  | -33 | 16 | 54 | 3.67 | 21 |
| Superior frontal gyrus | -25 | 55 | 13 | 3.60 | 50 |
| Supramarginal gyrus | 62 | -54 | 37 | 3.55 | 101 |
|  | -61 | -57 | 36 | 3.52 | 32 |
| Fusiform gyrus | -59 | -53 | -19 | 3.54 | 103 |
| Parahippocampal gyrus | -15 | -36 | 4 | 3.32 | 25 |
