# Supplementary material for: ANS: Aberrant Neurodevelopment of the Social Cognition Network in Adolescents with Autism Spectrum Disorders
Source: PLoS One. 2011 Apr 26;6(4):e18905. doi: 10.1371/journal.pone.0018905 (PMC3082537; doi:10.1371/journal.pone.0018905)
Supplement: Table S7 — Interaction effects of age by group in regional gray matter volume. (DOCX) [file pone.0018905.s007.docx]

**Table S7: Interaction effects of age by group in regional gray matter volume**

|  | **Peak coordinate** | | | ***Z*_≡_ score** | **Cluster size (mm^3^) (*P* < 0.001)** |
| --- | --- | --- | --- | --- | --- |
| **Anatomical location** | **x** | **y** | **z** |  |  |
| **TDC > ASD** |  |  |  |  |  |
| **Inferior parietal lobule** | **54** | **-30** | **44** | **3.81** | **84** |
| **Posterior cingulate** | **20** | **-63** | **12** | **3.31** | **40** |
| **ASD > TDC** |  |  |  |  |  |
| **Middle temporal gyrus** | **53** | **1** | **-19** | **3.58** | **88** |
| **Superior temporal gyrus** | **44** | **21** | **-25** | **3.50** | **334** |
| **Inferior temporal gyrus** | **42** | **-1** | **-38** | **3.49** | **58** |
