# Supplementary material for: ANS: Aberrant Neurodevelopment of the Social Cognition Network in Adolescents with Autism Spectrum Disorders
Source: PLoS One. 2011 Apr 26;6(4):e18905. doi: 10.1371/journal.pone.0018905 (PMC3082537; doi:10.1371/journal.pone.0018905)
Supplement: Table S8 — Interaction effects of age by subgroup (Autism vs. Asperger's syndrome) in regional gray matter volume. (DOCX) [file pone.0018905.s008.docx]

**Table S8: Interaction effects of age by subgroup (Autism vs. Asperger’s syndrome) in regional gray matter volume**

|  | **Peak coordinate** | | | ***Z*** | **Cluster size (mm^3^) (*P* < 0.001)** |
| --- | --- | --- | --- | --- | --- |
| **Anatomical location** | **x** | **y** | **z** |  |  |
| **Asperger syndrome > Autism** | | | | | |
| **Medial frontal gyrus** | -7 | 57 | 11 | 3.76 | 46 |
| **Lingual gyrus** | 19 | -74 | -4 | 3.67 | 124 |
| **Middle frontal gyrus** | -25 | 62 | 19 | 3.52 | 80 |
| **Middle frontal gyrus** | 45 | 12 | 37 | 3.51 | 63 |
| **Precentral gyrus** | 27 | -26 | 59 | 3.44 | 16 |
| **Middle occipital gyrus** | 38 | -80 | -8 | 3.33 | 11 |
| **Inferior frontal gyrus** | 54 | 39 | 2 | 3.3 | 13 |
| **Middle frontal gyrus** | -48 | 47 | -6 | 3.19 | 12 |
| **Autism > Asperger syndrome** | | | | | |
| **Postcentral gyrus** | 43 | -20 | 54 | 4.17 | 549 |
| **Middle temporal gyrus** | -41 | -65 | 21 | 47 | 147 |
| **Superior temporal gyrus** | -49 | 3 | -3 | 3.84 | 38 |
| **Parahippocampal gyrus** | -28 | -51 | -6 | 3.78 | 307 |
| **Fusiform gyrus** | -34 | -45 | -9 | 3.68 |  |
| **Parahippocampal gyrus** | -13 | -34 | 1 | 3.77 | 108 |
| **Fusiform gyrus** | 34 | -49 | -11 | 3.65 | 127 |
| **Middle temporal gyrus** | 53 | -65 | 18 | 3.61 | 212 |
| **Superior temporal gyrus** | 34 | 6 | -26 | 3.47 | 17 |
| **Uncus** | 29 | -17 | -30 | 3.25 | 28 |
| **Inferior temporal gyrus** | -60 | -23 | -19 | 3.23 | 13 |
