# Supplementary material for: ANS: Aberrant Neurodevelopment of the Social Cognition Network in Adolescents with Autism Spectrum Disorders
Source: PLoS One. 2011 Apr 26;6(4):e18905. doi: 10.1371/journal.pone.0018905 (PMC3082537; doi:10.1371/journal.pone.0018905)
Supplement: Table S9 — Interaction effects of age by group in regional gray matter concentration. (DOCX) [file pone.0018905.s009.docx]

**Table S9: Interaction effects of age by group in regional gray matter concentration**

|  | **Peak coordinate** | | | ***Z*_≡_ score** | **Cluster size (mm^3^) (*P* < 0.001)** |
| --- | --- | --- | --- | --- | --- |
| **Anatomical location** | **x** | **y** | **z** |  |  |
| **TDC > ASD** |  |  |  |  |  |
| **Inferior parietal lobule** | **-56** | **-58** | **38** | **3.22** | **84** |
| **ASD > TDC** |  |  |  |  |  |
| **Middle temporal gyrus** | **54** | **6** | **-37** | **3.48** | **41** |
| **Superior temporal gyrus** | **54** | **-55** | **16** | **3.24** | **20** |
| **Lingual gyrus** | **23** | **-57** | **1** | **3.22** | **26** |
