# Supplementary material for: ANS: Aberrant Neurodevelopment of the Social Cognition Network in Adolescents with Autism Spectrum Disorders
Source: PLoS One. 2011 Apr 26;6(4):e18905. doi: 10.1371/journal.pone.0018905 (PMC3082537; doi:10.1371/journal.pone.0018905)
Supplement: Table S10 — Interaction effects of age by group in regional white matter volume. (DOCX) [file pone.0018905.s010.docx]

**Table S10: Interaction effects of age by group in regional white matter volume**

|  | **Peak coordinate** | | | ***Z*** | **Cluster size (mm^3^) (*P* < 0.001)** |
| --- | --- | --- | --- | --- | --- |
| **Anatomical location** | **x** | **y** | **z** |  |  |
| **TDC > ASD** |  |  |  |  |  |
| **No Positive Correlation** |  |  |  |  |  |
| **TDC < ASD** |  |  |  |  |  |
| **Precuneus** | 22 | -57 | 40 | 3.82 | 260 |
| **Superior frontal gyrus** | 28 | 11 | 51 | 3.33 | 39 |
| **Middle frontal gyrus** | -28 | 26 | 36 | 3.25 | 32 |
